# Low Burden Strategies Are Needed to Reduce Smoking in Rural Healthcare Settings: A Lesson from Cancer Clinics

**DOI:** 10.3390/ijerph17051728

**Published:** 2020-03-06

**Authors:** Alex T. Ramsey, Timothy B. Baker, Giang Pham, Faith Stoneking, Nina Smock, Graham A. Colditz, Aimee S. James, Jingxia Liu, Laura J. Bierut, Li-Shiun Chen

**Affiliations:** 1 Department of Psychiatry, Washington University School of Medicine, St. Louis, MO 63110, USA; g.pham@wustl.edu (G.P.); faiths@wustl.edu (F.S.); smockn@wustl.edu (N.S.); laura@wustl.edu (L.J.B.); li-shiun@wustl.edu (L.-S.C.); 2 Alvin J. Siteman Cancer Center, Barnes-Jewish Hospital, Washington University School of Medicine, St. Louis, MO 63110, USA; colditzg@wustl.edu (G.A.C.); aimeejames@wustl.edu (A.S.J.); esther@wustl.edu (J.L.); 3 Department of Medicine, University of Wisconsin School of Medicine and Public Health, Madison, WI 53726, USA; tbb@ctri.wisc.edu; 4 Division of Public Health Sciences, Department of Surgery, Washington University School of Medicine, St. Louis, MO 63110, USA

**Keywords:** tobacco use, smoking cessation treatment, cancer care, rural, implementation strategies, electronic health record, decision support, health disparities

## Abstract

Rural populations face significant smoking-related health disparities, such as a higher prevalence of lung cancer and cancer mortality, higher prevalence of smoking, and lower likelihood of receiving cessation treatment than urban counterparts. A significant proportion of health disparities in rural populations could be eliminated with low-barrier, easy-access treatment delivery methods for smoking cessation. In this study, we assessed treatment engagement among patients in rural and urban settings. Then, we examined the effect of an electronic health record-based smoking cessation module on patient receipt of evidence-based cessation care. As part of a quality improvement project, we retrospectively observed 479,798 unique patients accounting for 1,426,089 outpatient clinical encounters from June 2018–March 2019 across 766 clinics in the greater St. Louis, southern Illinois, and mid-Missouri regions. Smoking prevalence was higher in rural versus urban clinics (20.7% vs. 13.9%, 6.7% [6.3, 7.1], odds ratio = 1.6 [1.6, 1.6], *p* < 0.0001), and yet rural smokers were nearly three times less likely than their urban counterparts to receive any smoking cessation treatment after adjusting for patients clustering within clinics (9.6% vs. 25.8%, −16.2% [−16.9, −15.5], odds ratio = 0.304 [0.28, 0.33], *p* < 0.0001). Although not yet scaled up in the rural setting, we examined the effects of a low-burden, point-of-care smoking module currently implemented in cancer clinics. After adjusting for patient clustering within clinics, patients were more likely to receive smoking treatment in clinics that implemented the module versus clinics that did not implement the module (31.2% vs. 17.5%*,* 13.7% [10.8, 16.6], odds ratio = 2.1 [1.8, 2.6], *p* < 0.0001). The point-of-care treatment approach offers a promising solution for rural settings, both in and outside the context of cancer care.

## 1. Introduction

Smoking leads to multiple health problems [1,2], including cancer [3]. Public health surveillance has identified geographical differences in smoking prevalence as well as smoking-related morbidity and mortality between urban and rural populations [4,5,6,7,8]. Rural populations have significantly higher prevalence of smoking compared to their urban counterparts [4,5] and consume a higher number of cigarettes per day [6]. Additionally, despite an overall reduction in cancer diagnoses [7], rural populations experience a higher likelihood of lung cancer diagnosis and death than urban populations—a pattern likely tied to the elevated prevalence of smoking in rural settings [3].

Although overall smoking prevalence has decreased in the last 10 years [9], the proportion of smokers in rural populations has remained stable or even increased [10,11], emphasizing the need for further efforts to assess smoking cessation treatment access within these communities. Dedicated attention from researchers, practitioners, and policy-makers is needed to address rural health and healthcare inequalities and enhance the reach of treatment into rural communities.

Generally, rural populations face multiple barriers to treatment, such as decreased access to clinics due to travel distances [12,13], reduced access to transportation [14], and lack of insurance [15,16], highlighting the need for cessation initiatives within these communities that address these barriers. For instance, although referral to tobacco treatment specialists is the prevailing model of cessation care in healthcare settings [17,18,19,20,21], this traditional approach is likely to encounter significant challenges in rural settings. This model assumes the presence of resources that are not widely available in rural communities: the availability of specialists in resource-limited settings, consistent provider referrals, and patients’ ability to travel and pay for specialist visits. Rural outpatient settings, including both oncology and primary care clinics, are in a unique position to assess smoking and provide cessation services [22,23]; furthermore, patients prefer that their medical providers include these services [24,25]. A better understanding of how patients in rural communities interact with their healthcare providers regarding smoking can help develop targeted interventions within clinics servicing these areas to capitalize on these encounters and provide cessation services.

The purpose of this study was to address current knowledge gaps on rural health and healthcare inequalities related to smoking and cessation treatment. Therefore, the present study utilized electronic health record (EHR) data from a large healthcare system covering clinics in Missouri and Illinois to assess differences in smoking prevalence and cessation treatment between urban and rural clinics. Given the unique challenges and documented treatment gaps in rural clinics, we also explored the effects of a low-burden, point-of-care smoking module that had been implemented in cancer clinics and was potentially scalable to rural settings. Therefore, this study examined the EHR-enabled smoking cessation module (ELEVATE) [26], a system-level implementation strategy designed to improve delivery of evidence-based care, and its effects on treatment access. The present study examines the following questions: (1) whether smoking prevalence differs between rural and urban clinics, (2) whether treatment engagement differs between rural and urban clinics, and (3) whether patients were more likely to receive cessation treatment in cancer clinics that had implemented versus clinics that had not yet implemented the smoking cessation module.

## 2. Materials and Methods

### 2.1. Design and Setting

This research was conducted as part of the National Cancer Institute (NCI) Cancer Moonshot program through the Cancer Center Cessation Initiative (C3I) and contributes to a larger effort to build and sustain a smoking cessation program that routinely provides cessation care for cancer patients [26]. This study is a cross-sectional, retrospective quality improvement project which aims to examine smoking prevalence among all patients and smoking cessation treatment engagement among current smokers who visited an outpatient clinic within a large Midwestern healthcare system. Patients received care in 766 clinics serving the greater St. Louis, southern Illinois, and mid-Missouri regions. Among the 766 clinics, 693 are located in urban and 73 are located in rural areas. There was no patient overlap in these urban and rural clinics. We identified the counties of the clinics using Google Earth Pro and classified them into six levels based on the CDC’s 2013 National Center for Health Statistics Urban–Rural Classification Scheme for Counties [27]. Urban clinics consisted of the first four levels of the Metropolitan Statistical Areas, which include the large central metro, large fringe metro, medium metro, and small metro. Rural clinics consisted of two nonmetropolitan statistical areas: micropolitan and noncore. We classified the clinic locations as urban or rural on 16 August 2019; we categorized clinic instead of patient home addresses to more directly examine the setting in which outpatient clinical encounters occurred.

To examine the association between exposure to the ELEVATE smoking cessation module and smoking treatment engagement, we examined patients seen in 45 cancer clinics, comparing the subset who visited clinics which had implemented the module (n = 29 clinics) versus the subset who visited clinics that had not yet implemented the module (n = 16 clinics). These clinics included Medical Oncology, Surgical Oncology, Radiation Oncology, Blood and Marrow Transplant, Gynecological Oncology, Hematology and Oncology, and Pediatric Hematology and Oncology. Implementation of the ELEVATE module was based on system organization under Epic Beacon, the medical oncology workflow system. Therefore, the module group primarily comprised medical oncology clinics, and the non-module group comprised various other oncology clinics. Honest data brokers unaffiliated with the current study performed a retrospective EHR search of patient evaluation and management encounters beginning at system-wide EHR transition to Epic on 2 June 2018 and ending on 31 March 2019.

### 2.2. ELEVATE Epic Module

We developed the ELEVATE Epic module [26] as a low-burden, point-of-care implementation strategy to systematically facilitate smoking assessment and treatment as part of every in-person clinical encounter. The ongoing purpose of the smoking module is to improve efficiency and efficacy by integrating a variation of the “5 As” framework into an EHR-assisted module. The module prompts the medical assistant or nurse to ask patients about smoking, advise cessation with a brief script built into the EHR, and assess their willingness to quit and to use phone, text, or app-based counseling. The module then provides the medical assistant or nurse with decision support to assist patient access to cessation options such as phone, text, or app-based counseling or cessation medications; for instance, the nurse can propose an order for nicotine replacement therapy in the EHR for the physician to sign. The module arranges for continued treatment and follow-up at the point of care, thus bypassing the conventional specialist referral model and enabling the clinical care team to provide assessment and treatment within one visit. Subsequently, the module facilitates smoking assessment at each following visit, and treatment options are re-offered every 90 days. The components of the ELEVATE Epic module are described fully elsewhere [26]. 

### 2.3. Sample

Our sample included adult (aged 18 years and older) patients (n = 479,798) with documented in-person outpatient clinical evaluation and management encounters (n = 1,426,089) completed between 2 June 2018 and 31 March 2019. Patients’ documented encounters were stratified by the location of the clinics to compare smoking prevalence and treatment engagement across urban and rural clinics. Clinics comprised by the data set were located across 18 counties in the greater St. Louis, southern Illinois, and mid-Missouri regions. Our sample included 124,688 medical outpatient visits from 50,250 patients who visited only rural clinics, 1,269,975 medical visits from 424,424 patients who visited only urban clinics, and 28,503 medical visits from 4,456 patients who visited both urban and rural clinics. When comparing smoking prevalence and treatment engagement between urban and rural clinics, we excluded the 668 patients whose encounters could not be located and the 4,456 patients who visited both urban and rural clinics. 

For the assessment of the ELEVATE smoking module, our sample included adult patients with documented in-person outpatient encounters at one of 45 cancer clinics completed between 2 June 2018 and 31 March 2019. Among patients who visited cancer clinics, 31,026 patients had encounters only at clinics that used the ELEVATE module, 10,188 patients had encounters only at clinics that did not, and 6,436 patients had encounters at both types of clinics. When comparing smoking prevalence and treatment engagement between clinics that used ELEVATE module and those that did not, we excluded the 6,436 patients who visited both clinics. Therefore, 41,214 patients were included in the analyses regarding the ELEVATE module.

### 2.4. Data Analysis

We analyzed both visit-level and patient-level data to capture smoking cessation treatment practices in a total of 1,426,089 patient visits made by 479,798 patients during the study time frame (2 June 2018–31 March 2019). We report smoking cessation practices at the patient level. We divided the sample into three groups—patients who only visited urban clinics, patients who only visited rural clinics, and patients who visited both urban and rural clinics—by filtering all encounters at urban and rural clinics. After excluding the third group, patients who visited both urban and rural clinics, we calculated smoking prevalence and treatment engagement for the other two groups based on their EHR documentation of one or more visits at each location. For example, a patient was coded as receiving cessation medication treatment if this patient received smoking cessation medication treatment during any visit in the study’s timeframe. 

For patients without smoking status assessment, if they received any smoking cessation treatment, they would be identified as smokers; that is, smoking prevalence was calculated for patients who were assessed for smoking (and documented as current smokers) or received any type of smoking cessation treatment. Treatment engagement was calculated for patients who identified as smokers. We used a similar approach when comparing these numbers between cancer clinics with and without the ELEVATE module. Statistical analysis included chi-squared tests and the generalized estimating equations methodology to control for the clustering of patients within clinics. All analyses were conducted using R version 3.5.3 for Microsoft Windows [28] and SAS software, Version 9.4m6 of the SAS system for Microsoft Windows [29] (copyright © 2020 SAS Institute Inc. SAS and all other SAS Institute Inc. product or service names are registered trademarks or trademarks of SAS Institute Inc., Cary, NC, USA).

## 3. Results

The demographic distribution of the sample used to compare urban and rural smoking prevalence and treatment engagement is presented in Table 1 (n = 474,674). The patient sample consisted of 59.5% females and 80.2% Caucasians, with ages ranging from 18 to 118. A total of 424,424 patients (89.4%) visited only urban clinics, and 50,250 patients (10.6%) visited only rural clinics. 

### 3.1. Smoking Prevalence Was Higher among Patients Seen in Rural Versus Urban Clinics

At the patient level, smoking prevalence was significantly higher in rural clinics (20.7%) compared to urban clinics (13.9%) (difference = 6.7%, 95% CI = [6.3–7.1], chi-squared = 1504.4, *p* < 0.0001) (Table 2). We observed similar findings when adjusting for the clustering of patients within clinics (Table 3). The likelihood of smoking was 1.6 times greater among patients in rural than in urban clinics after adjusting for patient clustering within clinics (odds ratio = 1.6 [1.6, 1.6], *p* < 0.0001).

### 3.2. Smoking Cessation Treatment Engagement Was Lower among Patients Seen in Rural Versus Urban Clinics

Alongside the higher smoking prevalence in rural clinics, a lower proportion of smokers received smoking treatment in rural clinics (9.6%) than in urban clinics (25.8%) (difference = −16.2% [−16.9, −15.5], chi-squared = 1214.5, *p* < 0.0001) (Table 2). Specific indicators of cessation treatment such as brief cessation advice, cessation medication treatment, and cessation counseling referral were found to be significantly lower in rural clinics than in urban clinics (Table 2). For example, only 3.2% of identified smokers seen in rural clinics were given brief cessation advice compared to 19.4% in urban clinics (difference = −16.2% [−16.7, −15.7], chi-squared = 1537.3, *p* < 0.0001). After adjusting for patient clustering within clinics, odds of cessation treatment engagement remained statistically significantly smaller in rural than in urban clinics (odds ratio = 0.304 [0.28, 0.33], *p* < 0.0001). Odds of engagement in specific types of cessation treatment were also lower for rural clinics, as shown in Table 3. 

### 3.3. Patients Were More Likely to Receive Cessation Treatment in Cancer Clinics That Had Implemented ELEVATE

The demographic distribution of the sample among cancer clinics used to compare smoking prevalence and treatment engagement between cancer clinics that implemented ELEVATE module and cancer clinics that did not implement ELEVATE module is presented in Table 4 (n = 41,214). The patient sample consisted of 64.6% females and 83.5% Caucasians, with ages ranging from 18 to 101. A total of 31,026 patients (75.3%) visited only cancer clinics that implemented ELEVATE module, and 10,188 patients (24.7%) visited only cancer clinics that did not implement ELEVATE module. 

We stratified smoking and cessation treatment engagement by ELEVATE module access to compare patients seen in cancer clinics with and without the module. Use of the module was associated with a higher proportion of patients receiving smoking cessation treatment. For example, in cancer clinics using the module, 31.2% of patients received any type of smoking cessation treatment compared to 17.5% in cancer clinics that did not use the module (difference = 13.7% [10.8, 16.6], chi-squared = 68.3, *p* ≤ 0.0001) (Table 5). Use of the module was also associated with higher proportions of patients receiving additional counseling offers (24.6% vs. 0.95%, difference = 23.6% [22.0, 25.3], chi-squared = 264.4, *p* < 0.0001), referral (2.9% vs. 0.0%, difference = 2.9% [2.3, 3.5], chi-squared = 27.0, *p* ≤ 0.0001), medication use (6.8% vs. 3.6%, difference = 3.2% [1.7, 4.7], chi-squared = 13.0, *p* = 0.00032) and brief advice (24.5% vs. 14.6%, difference = 9.9% [7.2, 12.6], chi-squared = 41.5, *p* ≤ 0.0001) (Table 5). When adjusted for patient clustering within clinics, the odds of smoking in cancer clinics that used the module was 1.3 times the odds of smoking in cancer clinics that did not use the module (odds ratio = 1.3 [1.2, 1.4], *p* < 0.0001). Treatment engagement remained higher in cancer clinics that used the module than in cancer clinics that did not use the module (odds ratio = 2.1 [1.8, 2.6], *p* < 0.0001). Cancer clinics that used the module were also more likely to deliver specific types of cessation treatment even after adjusting for patient clustering within clinics as shown in Table 6. We were not able to examine cancer clinic treatment engagement stratified by urban and rural settings as there were too few rural-based cancer clinics to compare rural versus urban cancer clinics.

## 4. Discussion

The present study addressed three primary research questions, finding that (1) smoking prevalence was higher among patients seen in rural versus urban clinics, (2) smoking cessation treatment engagement was lower among patients seen in rural versus urban clinics, and (3) patients were more likely to receive cessation treatment in cancer clinics that had implemented versus clinics that had not yet implemented the smoking cessation module. While previous research identified geographical differences in smoking prevalence using data collected from focus groups and national surveys [8,10,30,31], the present study used EHR data to further explore these discrepancies both by geographical location and among patients being seen in cancer clinics and highlighted gaps in smoking cessation treatment as well. Additionally, the present study implemented and assessed a low-burden, EHR-enabled intervention that assists with addressing low treatment engagement across geographical locations.

Among nearly 500,000 patients and 1,500,000 outpatient clinical encounters across 766 clinics, these data highlight a substantial disparity in smoking and cessation services in rural versus urban clinics. Smoking was highly prevalent at 20.7% among patients seen in rural clinics, compared to 13.9% in urban clinics, and yet urban smokers were about three times more likely to receive smoking treatment (by percentage) than rural smokers. This finding aligns with prior research showing that rural residents are more likely to smoke [8,10] and yet less likely to receive smoking treatment compared to urban and suburban residents [32]. 

The current findings support prior research by demonstrating that the smoking module increases the receipt of evidence-based smoking cessation treatment among cancer patients [26]. These data indicate that smoking cessation treatment engagement was significantly higher in cancer clinics that had implemented the smoking module (31.2%) versus cancer clinics that had not yet implemented the module (17.5%) (Table 5). Current smokers who visited clinics with the smoking module were more likely to receive cessation care than those from clinics without the module. This is of considerable clinical relevance, as cancer patients are too infrequently offered and engage in smoking cessation treatment [22,23]. 

Although prior research has reported that brief advice may be delivered in more than half of clinical encounters in oncology and primary care settings [33,34,35], the documentation of brief advice in the EHR within a large healthcare system is not well-known and likely to be highly variable across clinics and providers. We found that treatment engagement in both urban and rural clinics was considerably lower than these previous estimates, and we have strengthened this brief advice component by providing a standardized script for providers and integrating brief advice and other intervention components into the regular workflow. It is worth noting that the module did not appear to stimulate medication orders or additional counseling referrals to the same degree as it did for brief advice and offers of additional counseling. Despite enhancing the decision support tools for providers, the module may not have addressed important barriers, including established practices of medication prescribing, patient willingness to take medications, and misalignment of perspectives between patients and providers regarding patient interest in quitting [36]. Further, the module had not been optimized to more easily refer patients; thus, referral to additional counseling rates observed in the present study are similar to those in previous literature [37,38,39]. Optimizing the module with a closed-loop referral system could enhance referral to additional counseling, and addressing prescriber barriers could increase the number of medication prescriptions. Nevertheless, there may be substantial value in repeated offers of smoking cessation resources.

The current study has several limitations. Due to the cross-sectional design, there were no pre-test measures to establish equivalence between the clinics with and without the ELEVATE module. The lack of randomization does not allow for strong inferences regarding the effects of ELEVATE. In addition, possible interactions between rural versus urban and ELEVATE effects cannot be accurately determined at this time. Although we intended to examine cancer clinic treatment engagement broken down by urban and rural settings, there were too few clinics in the rural areas to permit this. When ELEVATE implementation is implemented across more clinics, we can test whether it improves smoking cessation treatment engagement in rural areas, where improved cessation care is particularly needed. Fidelity, or adherence to protocol, is important as it ensures that efficacious interventions are delivered appropriately to increase the likelihood that they will be effective in real-world pragmatic settings; however, given the nature of this system-level quality improvement study, we were limited in our ability to assess protocol adherence. In addition, smoking cessation outcome data—which will be observed via the EHR documentation of former smoker status over the 6 month period subsequent to treatment engagement—were not examined in this study. Those effectiveness data will ultimately inform the broader public health impact of this work. Finally, by region of the US, smoking prevalence is highest in the Midwest (18.5%) [40], where the study population is based. The findings of this study may be representative of other areas in the Midwest but be distinct from other regions in the US where smoking prevalence is much lower.

Future research could leverage the current findings to prioritize the implementation and evaluation of strategies to facilitate the delivery of evidence-based cessation care in outpatient rural settings, including rural cancer clinics. An important and understudied area remains the identification and use of a pragmatic and selective set of implementation strategy components—team-based training, decision support systems, data-driven feedback reports, participatory approaches and systems modeling, to name a few—that effectively aid in the delivery of evidence-based cessation interventions. For example, ongoing performance feedback can decrease treatment gaps by enhancing the transparency of discrepancies between an individual provider’s or a clinic’s propensity to offer or provide treatment in relation to a relevant comparator (e.g., other providers, clinic, benchmark). The ELEVATE module evaluated here represents a promising strategy to scale-up to rural settings with modern EHR systems, as it enables point-of-care cessation support that does not require substantial resources beyond the embedded clinical care team. 

## 5. Conclusions

This large-scale observational study quantified the prevalence of smoking and cessation treatment engagement among patients who smoke in a Midwest-based healthcare system. Smoking prevalence was particularly high and treatment engagement was particularly low among patients seen in rural clinics, where consistently delivered smoking cessation programs are lacking yet urgently needed. In the cancer care setting, patients were more likely to receive smoking treatment in clinics that implemented the EHR-based ELEVATE module versus clinics that did not implement the module. The point-of-care treatment approach supported by ELEVATE offers a promising solution for rural settings, both in and outside of the context of cancer care. Including decision support within EHR systems has the potential to extend the reach of cessation treatment across rural settings. This approach allows cancer care and outpatient clinical care teams more broadly to reduce treatment gaps and offer evidence-based cessation support to more patients who smoke. 

## Figures and Tables

**Table 1:** Sample demographics (n = 474,674).

	n	%
Gender *		
Female	282,283	59.5
Male	192,197	40.5
Race **		
Caucasian	371,208	80.2
African American	72,575	15.7
Other	19,273	4.2
**Age (years)**		
18-41	120,056	25.3
42-57	121,564	25.6
58-68	116,960	24.6
69-118	116,094	24.5
Clinic Location ***		
Urban	424,424	89.4
Rural	50,250	10.6

* Documented gender was missing for 194 patients. ** Documented race was missing for 11,618 patients. *** In total, 668 patients with missing clinic locations and 4,456 patients who went to both urban and rural clinics were excluded from our study.

**Table 2:** Smoking prevalence and treatment among urban and rural clinics.

	Rural		Urban					
	N	%	N	%	Diff.	95% CI	Chi-sq	*p*
**Total Patients** *	**50,250**		**424,424**					
Assessment	47,196	93.9	375,350	88.4	5.5	5.3, 5.7	1382.1	<0.0001
Smoking **	9751	20.7	52,369	13.9	6.7	6.3, 7.1	1504.4	<0.0001
**Total Smokers**	**9751**		**52,369**					
Any Treatment ***	934	9.6	13,526	25.8	−16.2	−16.9, −15.5	1214.5	<0.0001
Brief Advice	310	3.2	10,139	19.4	−16.2	−16.7, −15.7	1537.3	<0.0001
Medication	639	6.6	3929	7.5	−0.9	−1.5, −0.403	10.7	0.001
Additional Counseling Offer	161	1.7	1377	2.6	−1.0	−1.3, −0.68	32.2	<0.0001
Additional Counseling Referral ****	16	0.16	191	0.36	−0.20	−0.302, −0.099	9.4	0.002

* Out of 73 clinics at rural location, five clinics (6.8%) were cancer clinics. Out of 693 clinics at urban location, 40 clinics (5.8%) were cancer clinics. ** For patients without smoking status assessment, if they received any smoking cessation treatment, they would be identified as smokers. Therefore, smoking prevalence in rural and urban clinics were calculated for patients who were assessed and documented as smokers or received any type of smoking cessation treatment (n = 47,220 and 375,561). *** Any treatment is defined as patients receiving medication, brief advice given, or additional counseling was referred. **** For rural clinics, additional counseling referral (n = 16) consisted of engagement in Quitline referral (n = 10), QuitGuide or QuitStart app (n = 2), SmokefreeTXT (n = 2), and already referred at time of visit (n = 2). For urban clinics, additional counseling referral (n = 191) consisted of engagement in Quitline referral (n = 75), QuitGuide or QuitStart app (n = 14), SmokefreeTXT (n = 43), Quitline and app referral (n = 1), Quitline and SmokefreeTXT (n = 8), SmokefreeTXT and app (n = 3), and already referred at time of visit (n = 47).

**Table 3:** Results of generalized estimating equations analyses predicting smoking prevalence and treatment engagement in urban and rural clinics adjusting for the clustering of patients within clinics.

Outcome	Predictor	%	Odds Ratio	95%CI	*p*
**Total Patients (N = 474,674)**					
Assessment					
	Urban	88.4	Reference	--	--
	Rural	93.9	2.02	1.9, 2.1	<0.0001
Smoking *					
	Urban	13.9	Reference	--	--
	Rural	20.7	1.6	1.6, 1.6	<0.0001
**Total Smokers (N = 62,120)**					
Any Treatment **					
	Urban	25.8	Reference	--	--
	Rural	9.6	0.304	0.28, 0.33	<0.0001
Brief Advice					
	Urban	19.4	Reference		
	Rural	3.2	0.14	0.12, 0.15	<0.0001
Medication					
	Urban	7.5	Reference	--	--
	Rural	6.6	0.86	0.79, 0.94	0.001
Additional Counseling Offer					
	Urban	2.6	Reference	--	--
	Rural	1.7	0.62	0.53, 0.73	<0.0001
Additional Counseling Referral					
	Urban	0.36	Reference	--	--
	Rural	0.16	0.45	0.27, 0.75	0.002

* For patients without smoking status assessment, if they received any smoking cessation treatment, they would be identified as smokers. Therefore, smoking prevalence in rural and urban clinics were calculated for patients who were assessed and documented as smokers or received any type of smoking cessation treatment (n = 422,781). ** Any treatment is defined as patients receiving medication, brief advice given, or additional counseling was referred.

**Table 4:** Sample demographics among patients in cancer clinics (n = 41,214).

	n	%
**Gender**		
Female	26,641	64.6
Male	14,573	35.4
Race *		
Caucasian	34,143	83.5
African American	5647	13.8
Other	1079	2.6
**Age (years)**		
18-53	10,514	25.5
54-63	10,120	24.6
64-72	10,578	25.7
73-101	10,002	24.3
**ELEVATE Module**		
Yes	31,026	75.3
No	10,188	24.7
Clinic Location **		
Urban	40,042	97.2
Rural	1114	2.7
Both	58	0.1

* Documented race was missing for 345 patients. ** Patients who visited cancer clinics were divided into three groups based on the clinics’ locations: patients who visited only urban clinics, patients who visited only rural clinics, and patients who visited both urban and rural clinics.

**Table 5:** Smoking prevalence and treatment in cancer clinics by use of the ELEVATE module.

	Module		No Module					
	N	%	N	%	Diff.	95% CI	Chi-sq	*p*
Total Patients *	**31,026**		**10,188**					
Assessment	26,350	84.9	9170	90.0	−5.1	−5.8, −4.4	165.7	<0.0001
Smoking **	3445	13.1	952	10.4	2.7	1.9, 3.4	45.1	<0.0001
**Total Smokers**	**3445**		**952**					
Any Treatment ***	1076	31.2	167	17.5	13.7	10.8, 16.6	68.3	<0.0001
Brief Advice	844	24.5	139	14.6	9.9	7.2, 12.6	41.5	<0.0001
Medication	234	6.8	34	3.6	3.2	1.7, 4.7	13.0	0.00032
Additional Counseling Offer	847	24.6	9	0.95	23.6	22.0, 25.3	264.4	<0.0001
Additional Counseling Referral ****	100	2.9	0	0.0	2.9	2.3, 3.5	27.0	<0.0001

* Out of 31,026 patients who visited cancer clinics that used the module, 29,854 patients (97.2%) only visited urban cancer clinics, 1,114 patients (3.6%) only visited rural cancer clinics, and 58 patients (0.2%) visited both urban and rural cancer clinics. There were no cancer clinics that did not use the module at rural location in this sample. ** For patients without smoking status assessment, if they received any smoking cessation treatment, they would be identified as smokers. Therefore, smoking prevalence in cancer clinics that used the module and cancer clinics that did not use the module were calculated for patients who were assessed and documented as smokers or received any type of smoking cessation treatment (n = 26,372 and 9,176). *** Any treatment is defined as patients receiving medication, brief advice given, or additional counseling was referred. **** For clinics with the module, additional counseling referral (n = 100) consisted of engagement in Quitline referral (n = 49), QuitGuide or QuitStart app (n = 5), SmokefreeTXT (n = 17), Quitline and SmokefreeTXT (n = 3), and already referred at time of visit (n = 26). For clinics without the module, no patients received additional counseling referral.

**Table 6:** Results of generalized estimating equations analyses predicting smoking prevalence and treatment engagement among patients in cancer clinics that used the module and cancer clinics that did not use the module adjusting for the clustering of patients within clinics.

Outcome	Predictor	%	Odds Ratio	95%CI	*p*
**Total Patients (N = 41,214)**					
Assessment					
	No module	90.0	Reference	--	--
	Module	84.9	0.63	0.58, 0.67	<0.0001
Smoking *					
	No module	10.4	Reference	--	--
	Module	13.1	1.3	1.2, 1.4	<0.0001
**Total Smokers (N = 4397)**					
Any Treatment **					
	No module	17.5	Reference	--	--
	Module	31.2	2.1	1.8, 2.6	<0.0001
Brief Advice					
	No module	14.6	Reference	--	--
	Module	24.5	1.9	1.6, 2.3	<0.0001
Medication					
	No module	3.6	Reference	--	--
	Module	6.8	2.0	1.4, 2.8	0.0003
Additional Counseling Offer					
	No module	0.95	Reference	--	--
	Module	24.6	34.2	17.6, 66.1	<0.0001
Additional Counseling Referral ***					
	No module	0.0	Reference	--	--
	Module	2.9	--	--	--

* For patients without smoking status assessment, if they received any smoking cessation treatment, they would be identified as smokers. Therefore, smoking prevalence in cancer clinics that used the module and cancer clinics that did not use the module were calculated for patients who were assessed and documented as smokers or received any type of smoking cessation treatment (n = 35,548). **Any treatment is defined as patients receiving medication, brief advice given, or additional counseling was referred. *** Since there were no patients referred for additional counseling in cancer clinics that use the module, we could not get an estimate for this indicator of treatment engagement.

## References

[1] CDC Tobacco-Related Mortality. https://www.cdc.gov/tobacco/data_statistics/fact_sheets/health_effects/tobacco_related_mortality/index.htm.

[2] CDC Health Effects of Cigarette Smoking. https://www.cdc.gov/tobacco/data_statistics/fact_sheets/health_effects/effects_cig_smoking/index.htm#ref.

[3] CDC Tobacco and Cancer. https://www.cdc.gov/cancer/tobacco/index.htm.

[4] Doogan N.J., Roberts M.E., Wewers M.E., Stanton C.A., Keith D.R., Gaalema D.E., Kurti A.N., Redner R., Cepeda-Benito A., Bunn J.Y. (2017). A growing geographic disparity: Rural and urban cigarette smoking trends in the United States. Prev. Med..

[5] Nighbor T.D., Doogan N.J., Roberts M.E., Cepeda-Benito A., Kurti A.N., Priest J.S., Johnson H.K., Lopez A.A., Stanton C.A., Gaalema D.E. (2018). Smoking prevalence and trends among a U.S. national sample of women of reproductive age in rural versus urban settings. PloS ONE.

[6] Roberts M.E., Doogan N.J., Kurti A.N., Redner R., Gaalema D.E., Stanton C.A., White T.J., Higgins S.T. (2016). Rural tobacco use across the United States: How rural and urban areas differ, broken down by census regions and divisions. Health Place.

[7] CDC (2017). First Report to Detail Cancer Differences and Mortality Gap between Rural and Urban Areas.

[8] CDC (2010). State-Specific Prevalence of Cigarette Smoking and Smokeless Tobacco Use Among Adults—United States, 2009.

[9] CDC (2019). Tobacco Product Use and Cessation Indicators Among Adults—United States, 2018.

[10] Vander Weg M.W., Cunningham C.L., Howren M.B., Cai X. (2011). Tobacco use and exposure in rural areas: Findings from the Behavioral Risk Factor Surveillance System. Addict. Behav..

[11] Doescher M.P., Jackson J.E., Jerant A., Gary Hart L. (2006). Prevalence and Trends in Smoking: A National Rural Study. J. Rural. Health.

[12] Douthit N., Kiv S., Dwolatzky T., Biswas S. (2015). Exposing some important barriers to health care access in the rural USA. Public Health.

[13] Deligiannidis K.E. (2017). Primary Care Issues in Rural Populations. Prim. Care.

[14] Arcury T.A., Preisser J.S., Gesler W.M., Powers J.M. (2005). Access to transportation and health care utilization in a rural region. J. Rural. Health.

[15] Casey M.M., Thiede Call K., Klingner J.M. (2001). Are rural residents less likely to obtain recommended preventive healthcare services?. Am. J. Prev. Med..

[16] Eberhardt M.S., Pamuk E.R. (2004). The Importance of Place of Residence: Examining Health in Rural and Nonrural Areas. Am. J. Public Health.

[17] Adsit R.T., Fox B.M., Tsiolis T., Ogland C., Simerson M., Vind L.M., Bell S.M., Skora A.D., Baker T.B., Fiore M.C. (2014). Using the electronic health record to connect primary care patients to evidence-based telephonic tobacco quitline services: A closed-loop demonstration project. Transl. Behav. Med..

[18] Sheffer C.E., Anders M., Brackman S.L., Steinberg M.B., Barone C. (2012). Tobacco intervention practices of primary care physicians treating lower socioeconomic status patients. Am. J. Med. Sci..

[19] Sheffer M.A., Baker T.B., Fraser D.L., Adsit R.T., McAfee T.A., Fiore M.C. (2012). Fax referrals, academic detailing, and tobacco quitline use: A randomized trial. Am. J. Prev. Med..

[20] Association for the Treatment of Tobacco Use and Dependence. Core Competencies For Evidence-based Treatment of Tobacco Dependence, April, 2005.

[21] Vander Weg M.W., Holman J.E., Rahman H., Sarrazin M.V., Hillis S.L., Fu S.S., Grant K.M., Prochazka A.V., Adams S.L., Battaglia C.T. (2017). Implementing smoking cessation guidelines for hospitalized Veterans: Cessation results from the VA-BEST trial. J. Subst. Abuse Treat..

[22] Fiore M.C., D’Angelo H., Baker T. (2019). Effective Cessation Treatment for Patients With Cancer Who Smoke—The Fourth Pillar of Cancer Care. JAMA Netw Open.

[23] Croyle R.T., Morgan G.D., Fiore M.C. (2019). Addressing a Core Gap in Cancer Care—The NCI Moonshot Program to Help Oncology Patients Stop Smoking. N. Engl. J. Med..

[24] Cluss P.A., Moss D. (2002). Parent attitudes about pediatricians addressing parental smoking. Ambul. Pediatr..

[25] Kviz F.J., Clark M.A., Hope H., Davis A.M. (1997). Patients’ perceptions of their physician’s role in smoking cessation by age and readiness to stop smoking. Prev. Med..

[26] Ramsey A.T., Chiu A., Baker T., Smock N., Chen J., Lester T., Jorenby D.E., Colditz G.A., Bierut L.J., Chen L.S. (2019). Care-paradigm shift promoting smoking cessation treatment among cancer center patients via a low-burden strategy, Electronic Health Record-Enabled Evidence-Based Smoking Cessation Treatment. Transl. Behav. Med..

[27] Ingram D.D., Franco S.J. (2014). 2013 NCHS Urban-Rural Classification Scheme for Counties.

[28] R Core Team (2019). R: A Language and Environment for Statistical Computing.

[29] SAS Institute Inc (2016). SAS Software for Windows, 9.4M6.

[30] CDC (2012). Current cigarette smoking among adults—United States, 2011. Morb. Mortal. Wkl. Rep..

[31] CDC Tobacco Use by Geographic Region. https://www.cdc.gov/tobacco/disparities/geographic/index.htm.

[32] Hutcheson T.D., Greiner K.A., Ellerbeck E.F., Jeffries S.K., Mussulman L.M., Casey G.N. (2008). Understanding smoking cessation in rural communities. J. Rural. Health.

[33] Bartsch A.-L., Härter M., Niedrich J., Brütt A.L., Buchholz A. (2016). A Systematic Literature Review of Self-Reported Smoking Cessation Counseling by Primary Care Physicians. PloS ONE.

[34] Kruger J., O’Halloran A., Rosenthal A. (2015). Assessment of compliance with US Public Health Service Clinical Practice Guideline for tobacco by primary care physicians. Harm Reduct. J..

[35] Simmons V.N., Litvin E.B., Unrod M., Brandon T.H. (2012). Oncology healthcare providers’ implementation of the 5A’s model of brief intervention for smoking cessation: Patients’ perceptions. Patient Educ. Couns..

[36] Chen L.-S., Baker T., Brownson R.C., Carney R.M., Jorenby D., Hartz S., Smock N., Johnson M., Ziedonis D., Bierut L.J. (2016). Smoking Cessation and Electronic Cigarettes in Community Mental Health Centers: Patient and Provider Perspectives. Community Ment. Health J..

[37] Kaufman A., Augustson E., Davis K., Finney Rutten L.J. (2010). Awareness and use of tobacco quitlines: Evidence from the Health Information National Trends Survey. J. Health Commun..

[38] Cummins S.E., Bailey L., Campbell S., Koon-Kirby C., Zhu S.H. (2007). Tobacco cessation quitlines in North America: A descriptive study. Tob. Control..

[39] Ossip-Klein D.J., McIntosh S. (2003). Quitlines in North America: Evidence base and applications. Am. J. Med. Sci..

[40] Jamal A., Phillips E., Gentzke A.S., Homa D.M., Babb S.D., King B.A., Neff L.J. (2018). Current Cigarette Smoking Among Adults—United States, 2016. MMWR. Morb. Mortal. Wkl. Rep..

